# Biomechanical comparative analysis of conventional pedicle screws and cortical bone trajectory fixation in the lumbar spine: An in vitro and finite element study

**DOI:** 10.3389/fbioe.2023.1060059

**Published:** 2023-01-19

**Authors:** Baoqing Pei, Yangyang Xu, Yafei Zhao, Xueqing Wu, Da Lu, Haiyan Wang, Shuqin Wu

**Affiliations:** ^1^ Beijing key laboratory for design and evaluation technology of advanced implantable & interventional medical devices, Beijing Advanced Innovation Center for Biomedical Engineering, School of Biological Science and Medical Engineering, Beihang University, Beijing, China; ^2^ Aerospace center hospital, Beijing, China; ^3^ School of Basic Medicine, Inner Mongolia Medical University, Hohhot, China; ^4^ School of Big Data and Information, Shanxi College of Technology, Shanxi, China

**Keywords:** biomechanical phenomena, finite element analysis, biomechanical tests, cortical bone trajectory screws, pedicle screws, hybrid screw strategy

## Abstract

Numerous screw fixation systems have evolved in clinical practice as a result of advances in screw insertion technology. Currently, pedicle screw (PS) fixation technology is recognized as the gold standard of posterior lumbar fusion, but it can also have some negative complications, such as screw loosening, pullout, and breakage. To address these concerns, cortical bone trajectory (CBT) has been proposed and gradually developed. However, it is still unclear whether cortical bone trajectory can achieve similar mechanical stability to pedicle screw and whether the combination of pedicle screw + cortical bone trajectory fixation can provide a suitable mechanical environment in the intervertebral space. The present study aimed to investigate the biomechanical responses of the lumbar spine with pedicle screw and cortical bone trajectory fixation. Accordingly, finite element analysis (FEA) and *in vitro* specimen biomechanical experiment (IVE) were performed to analyze the stiffness, range of motion (ROM), and stress distribution of the lumbar spine with various combinations of pedicle screw and cortical bone trajectory screws under single-segment and dual-segment fixation. The results show that dual-segment fixation and hybrid screw placement can provide greater stiffness, which is beneficial for maintaining the biomechanical stability of the spine. Meanwhile, each segment’s range of motion is reduced after fusion, and the loss of adjacent segments’ range of motion is more obvious with longer fusion segments, thereby leading to adjacent-segment disease (ASD). Long-segment internal fixation can equalize total spinal stresses. Additionally, cortical bone trajectory screws perform better in terms of the rotation resistance of fusion segments, while pedicle screw screws perform better in terms of flexion–extension resistance, as well as lateral bending. Moreover, the maximum screw stress of L4 cortical bone trajectory/L5 pedicle screw is the highest, followed by L45 cortical bone trajectory. This biomechanical analysis can accordingly provide inspiration for the choice of intervertebral fusion strategy.

## 1 Introduction

**FIGURE 1:**
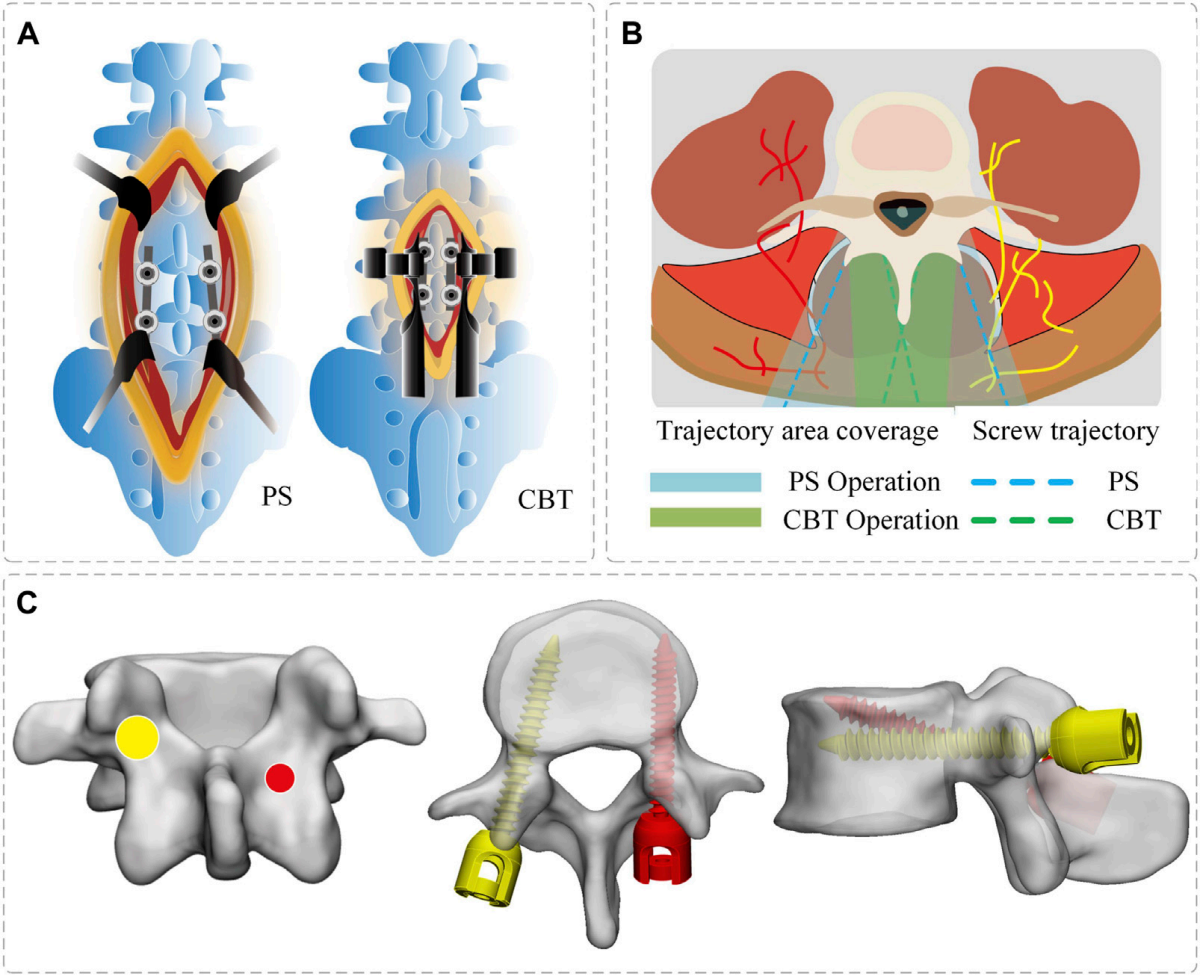
Schematic diagram of screw placement. **(A)** Surgical incision in PS/CBT group. **(B)** PS/CBT trajectory area coverage. **(C)** Position of the entry point and screw trajectory (PS—yellow, CBT—red). The screw entry point PS is located at the apex of the herringbone crest and CBT is located at the isthmus of the vertebral arch.

Numerous intervertebral fusion techniques have arisen as a result of the ongoing upgrading of internal fixation techniques. There are several intervertebral fusion techniques, since internal fixation techniques are always being updated. After short-segment fixation, ASD may emerge, necessitating the selection of yet another surgical strategy. The traditional trajectory for PS requires significant tissue dissection and muscle retraction, whereas the cortical bone trajectory (CBT) screw has the advantages of minimal muscle damage and the preservation of the superior facet joint (Qiu et al., 2022) (Figure 1). Many biomechanical studies have proved that the CBT technique provides greater pull-out strength, rigid insertion torque fixation, and a stable screw structure similar to traditional PS (Matsukawa et al., 2016; Sansur et al., 2016). Less research has been carried out on the impact of various screw implantation strategies on the stability and flexibility of the lumbar spine. The effects of numerous spinal fusion methods on spinal stiffness are still unclear, and research on the benefits and drawbacks of each method is still lacking. Physicians are curious to see whether combining traditional PS and CBT has benefits and how the unique mechanical features function. How to select the correct screw fixation technology to treat degenerative spinal conditions is the problem of the greatest concern for clinicians. For this reason, FEA and IVEs are frequently available.

For the treatment of lumbar instability and ADS surgery, a precise biomechanical analysis of the various surgical modalities is necessary to choose the best option for effective lumbar internal fixation. To define the intricate biomechanical characteristics of the lumbar spine, complementary methods include FEA and IVEs (Xu et al., 2013; Lu and Lu, 2019). In this study, the distribution pattern of the internal fixation system was explored, and the data were analyzed to explore the mechanical references for procedure selection in clinical practice. FEA and IVEs of the T12-S1 vertebral body were performed using different combinations of screw placement techniques to reveal the biomechanical differences between different implantation techniques. The main goals of this study were as follows: 1) to create a workable finite element model (FEM) of the lumbar spine; 2) to simulate screw placement techniques on the models; 3) to compare the stress distribution of the posterior screw–rod system under various screw placement techniques; 4) to create specimen models of various posterior screw–rod systems; and 5) to test the differences in joint mobility and stiffness values between the FEMs and the IVEs.

## 2 Materials and methods

The research was approved by the Science and Ethics Committee of the School of Biological Science and Medical Engineering at Beihang University (protocol code: BM20220087).

### 2.1 Grouping

**FIGURE 2:**
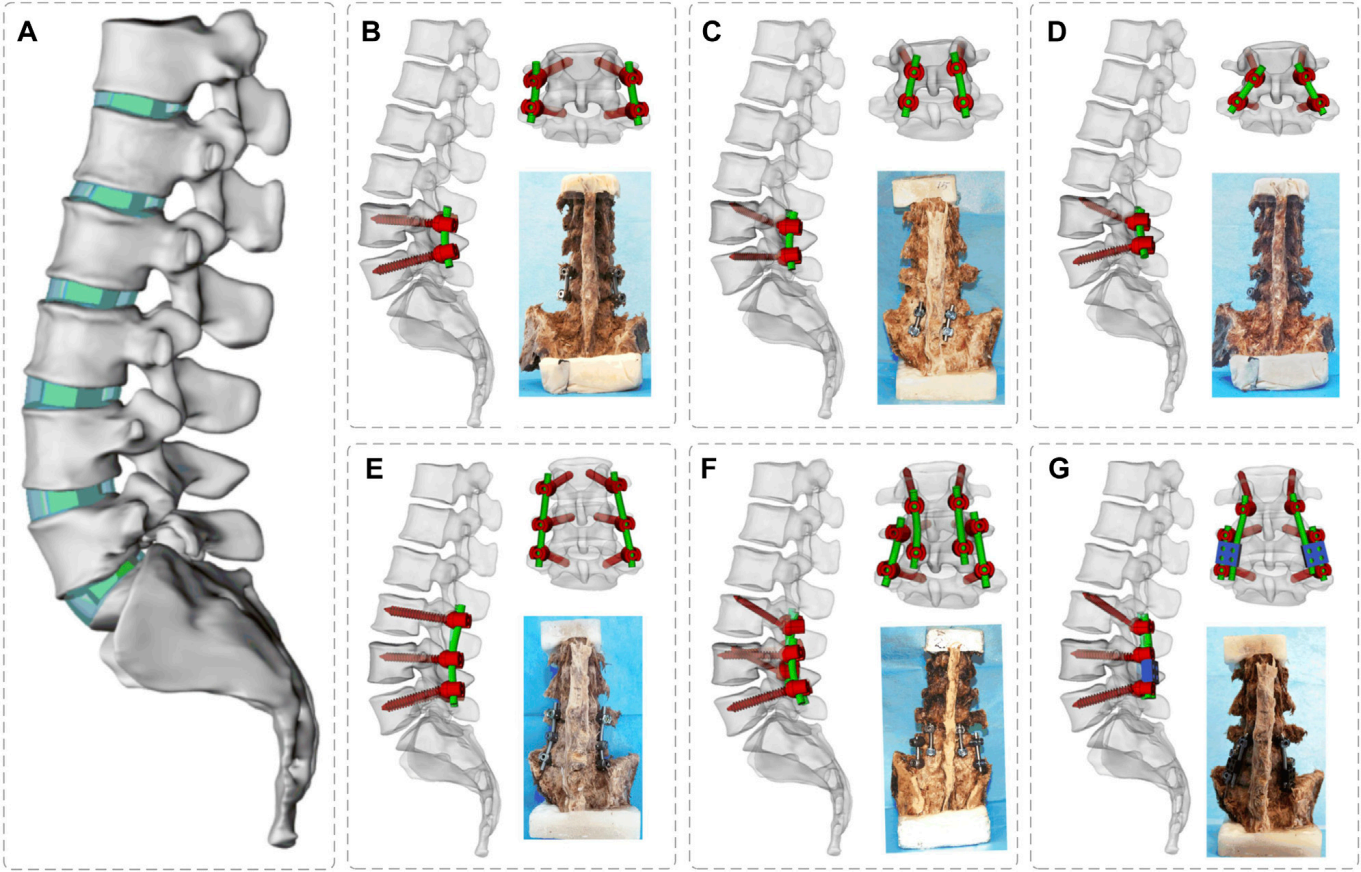
Three-dimensional simulation models and spine specimen models for posterior internal fixation of T12-S segment lumbar spine. **(A)** Intact model, **(B)** L45 PS model, **(C)** L45CBT model, **(D)** L4 CBT/L5 PS model, **(E)** L345 PS model, **(F)** L34CBT/L45PS model, and **(G)** L3 CBT-L45 PS model.

In this study, we established one intact model, three common clinical single-segment fusion groups, and three dual-segment fusion groups for the treatment of ASD, with a total of seven groups of models: 1) the intact model, 2) the L45PS model, 3) the L45CBT model, 4) the L4CBT/L5 PS model, 5) the L345 PS model, 6) the L34CBT/L45PS model, and 7) the L3CBT lateral connection L45PS (L3CBT-L45PS) model (Figure 2).

### 2.2 *In Vitro* specimen biomechanical experiments

#### 2.2.1 Sample selection and processing

Seven adult spine specimens (provided by Beijing Chaoyang Hospital of Capital Medical University) were selected, preserving CT imaging data without obvious imaging abnormalities, such as lumbar spine trauma, tumor, tuberculosis, scoliosis, lumbar spondylolisthesis, ischial cleft, and other diseases. Before the experiment, the specimens were wrapped in multiple layers of cling film and stored at a temperature of −20°C. The specimens were then thawed at a temperature of 4°C for 12–18 h. Using the T12-S1 section of the lumbar spine as the experimental sample, the muscles and soft tissues surrounding the vertebrae were removed in accordance with the anatomical structure, but the ligaments, tiny joints, and intervertebral discs were left in place. The specimens were kept moist with 0.9% saline throughout the testing procedure. The upper end of the L1 vertebral body and the caudal end of the S1 vertebral body was embedded in polymethylmethacrylate (PMMA) using a custom-made embedding cassette mounted on the testing device (Wang et al., 2020a). Screw placement was carried out by orthopedic surgeons from Beijing Chao-yang Hospital of Capital Medical University.

#### 2.2.2 Experimental methods and procedures

**FIGURE 3:**
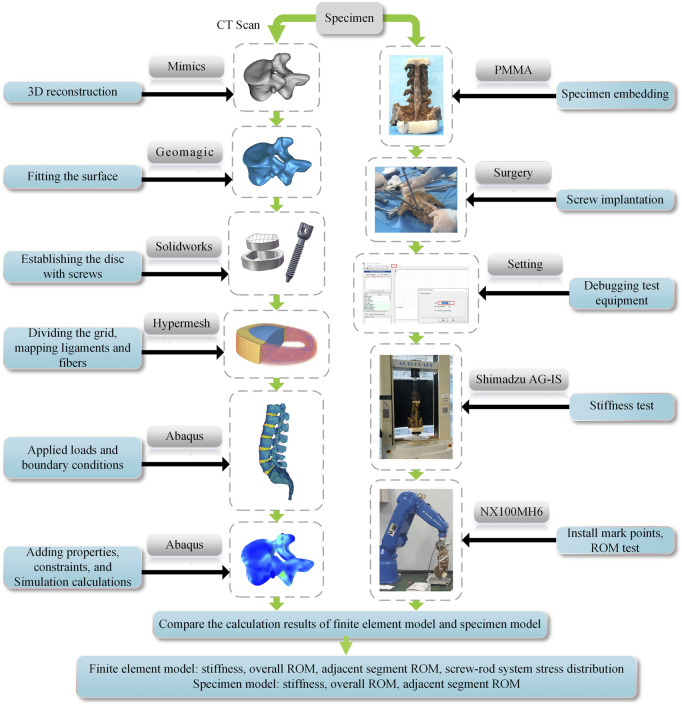
Flow chart of FEA and IVE.

The upper end of vertebra L1 is fixedly attached to the front end of a six-degree-of-freedom robotic arm (NX100MH6, Yaskawa Robotic Arm, Kitakyushu, Japan), and the tail vertebra S1 is embedded in a fixed base frame. A moment sensor (Gamma, ATI Industrial Automation, Ontario, Canada) is mounted on the head of the robot arm to record applied forces and moments and provide real-time feedback. The NDI system (Optotrak Certus, North Digital Ltd., Waterloo, Canada) captures the motion path of each vertebral segment by recording the position of several sets of marker points. Motion data acquisition utilizes the 3D spatial coordinate system differences of the NDI system to determine the relative motion position of the vertebral body. In this experiment, five marker points were fixed at T12, L1, L2, L3, L4, L5, and the base (reference point) for capturing the motion path of each segment during lumbar vertebral motion (Figure 3).

The experimental loading method was as follows: a Panjabi pure moment loading control protocol was used with a constant loading rate of 1.0°/s (Panjabi, 2003; Panjabi, 2007). In the experiment, the moment-controlled loading mode was used, and the loading mode procedure was set to 7.5°Nm of forwarding flexion, back extension, left and right lateral bending, and left and right axial rotation. This experiment procedure requires the constant spraying of saline on the specimen to maintain wetness and a room experimental temperature of 25°C.

### 2.3 Finite element analysis

#### 2.3.1 Finite element model establishing

High-resolution CT scans were performed on the specimens, and the obtained DICOM data were imported into Mimics 21.0 (Materialise, Leuven, Belgium) to obtain the T12-S1 vertebrae by threshold segmentation. To obtain a high-quality vertebral model, the model was processed in Geomagic Studio 2013 (Raindrop Geomagic Inc., Morrisville, NC, United States) for denoising, smoothing, and fitting the surface; the medullary nucleus and fibrous ring matrix were drawn in Solidworks (SolidWorks Corp., Waltham, MA, United States). The entire model was meshed and the ligament and annulus fibrosus fibers were created in Hypermesh (Altair Engineering Inc., Troy, MI, United States). The material properties, set boundaries, loading conditions, and computational conditions were defined, and the FEA was completed in Abaqus (Simulia, Providence, Rhode Island, United States) (Figure 3).

The bone tissue consists of cortical and cancellous elements. The intervertebral disc has a nucleus pulposus, a fibrous annulus matrix, and fibrous annulus fibrosus, which is divided into seven layers (Wang et al., 2019). The model includes the major lumbar ligaments: including the anterior longitudinal ligament (ALL), posterior longitudinal ligament (PLL), ligamentum flavum (FL), supraspinous ligament (SSL), interspinous ligament (ISL), intertransverse ligament (TL), and capsular ligament (FC). A 0.25 mm-thick cartilage layer was also added to the surface of each small joint and a 0.5 mm gap was created between the curved small joints (Caruso et al., 2018). PS size (diameter 6.5 mm, length 45 mm), CBT screw size (diameter 5 mm, length 35 mm), and connecting rod size (diameter 5.5 mm) were set. Tetrahedral meshing was performed for all vertebrae, intervertebral discs, articular cartilage, and screw–rod systems, and the material properties of each part of the model and the cross-sectional areas of ligaments and fibers are shown in Table 1.

**TABLE 1:** Material properties of the models.

Structure	Young’s modulus (MPa)	Poisson’s ratio	Cross-section area (mm^2^)
Cortical bone	Ex = 11,300, Ey = 11,300, Ez = 22,300	V_xy_ = 0.484, V_xz_ = 0.203, V_yz_ = 0.203	-
	G_X_ = 3,800, G_Y_ = 5,400, G_Z_ = 5,400		
Cancellous bone	Ex = 140, Ey = 140, Ez = 200	V_xy_ = 0.45, V_xz_ = 0.315, V_yz_ = 0.315	-
	G_X_ = 48.3, G_Y_ = 48.3, G_Z_ = 48.3		
ALL	7.8(<12.0%), 20.0(>12.0%)	0.40	63.7
PLL	10.0(<11.0%), 20.0(>11.0%)	0.30	20
SSL	8.0(<20.0%), 15.0(>20.0%)	0.30	70
ISL	10.0(<14.0%), 11.6(>14.0%)	0.30	70
LF	15.8(<6.2%), 19.5(>6.2%)	0.30	40
TL	10.0(<18.0%), 58.4(>18.0%)	0.30	1.8
CL	7.5(<25.0%), 32.9(>25.0%)	0.30	30
Nucleus pulposus	Hyperelastic, Mooney–Rivlin: C^10^ = 0.18, C^01^ = 0.045	-	-
Annulus fibrosus matrix	Hyperelastic, Mooney–Rivlin: C^10^ = 0.12, C^01^ = 0.03	-	-
Fiber	360–550	0.30	0.15
screw–rods system	110,000	0.28	-

ALL, anterior longitudinal ligament; PLL, posterior longitudinal ligament; SSL, supraspinal ligament; ISL, interspinous ligament; LF, ligamentum flavum; TL, transverse ligaments; CL, capsular ligament.

#### 2.3.2 Boundary conditions

**FIGURE 4:**
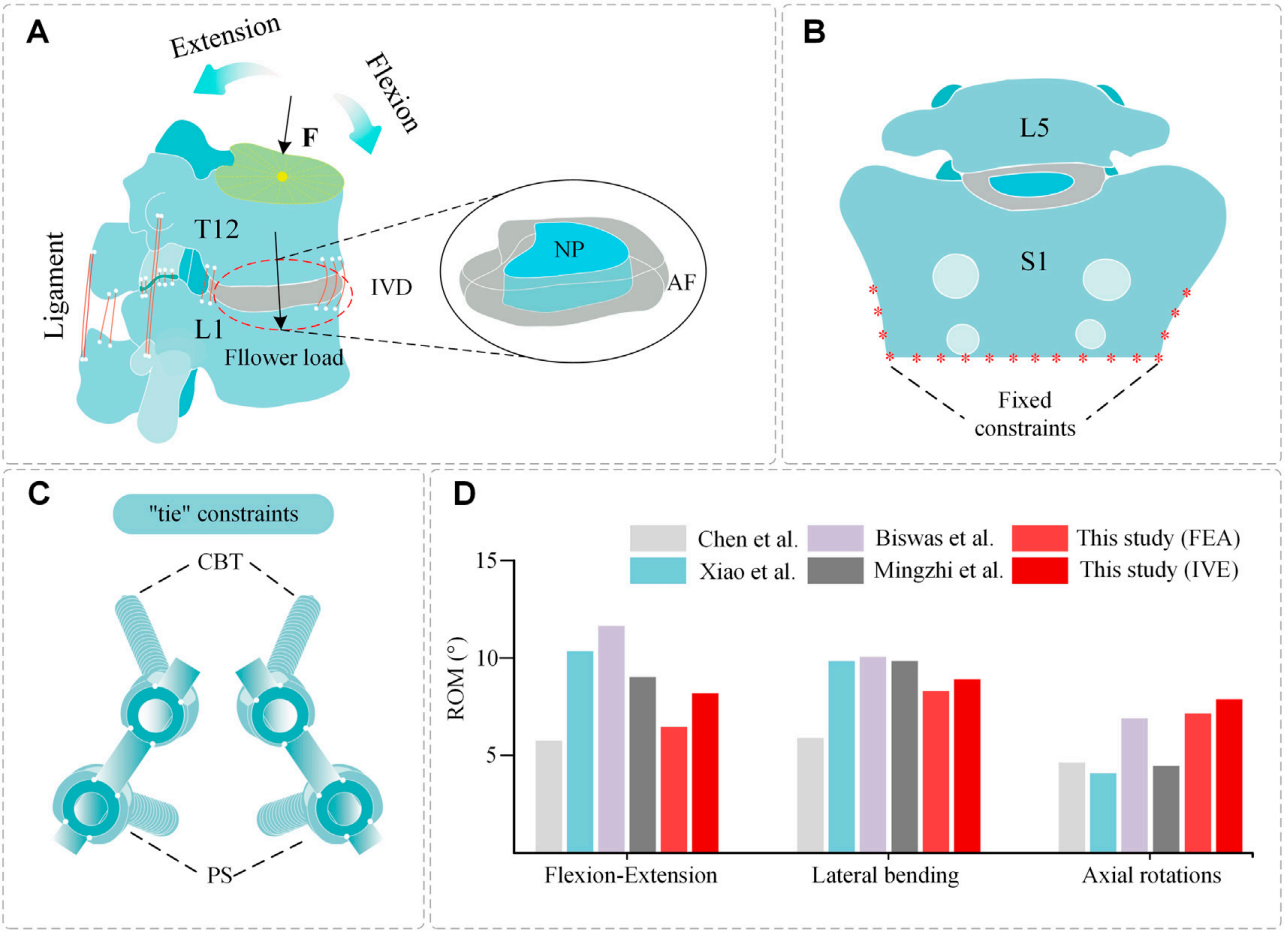
Spinal composition, constraint setting, and validation. **(A)** Spinal constraint setting: compression force F, follower load. **(B)** Spinal constraint setting: S1 fixed constraints. **(C)** Hybrid placement of screws and “tie” constrains. **(D)** Comparison of ROM, between this study and the results reported in the previous literature.

The local muscle force of the lumbar spine was provided by a follower load of 200 N (Rui et al., 2018), and a preload compression force of 400 N body weight was applied at the center of the upper endplate of T12 (Renner et al., 2007). The physical movements of flexion, extension, right and left lateral bending, and right and left axial rotation were replicated using a torque of 7.5 Nm (Alizadeh et al., 2013), small inter-articular contacts were set with a friction factor of 0.1 (Tsouknidas, 2015), and all remaining contacts were “tie” constraints. Fixation constraints were added at the sacrum (Figures 4A,B,C).

### 2.4 Validation of the models

The ROM of each segmental value measured by the model was compared with the experimental data reported in previous studies to validate the plausibility of the model. Mean ROM values were measured in each motion type and the results were compared with previously published biomechanical experiments and FEA (Chen et al., 2001; Xiao et al., 2012; Biswas et al., 2018; Song et al., 2021). The trends were consistent and there were no significant differences in the data, proving that the model was reasonable (Figure 4D).

## 3 Results

In this study, the following parameters were evaluated: 1) compressive stiffness in the T12-S segment; 2) overall ROM; 3) ROM of the adjacent segment; 4) ROM of each segment of T12-L5; and 5) Von Mises stress distribution of the screw–rod system.

### 3.1 Stiffness

**FIGURE 5:**
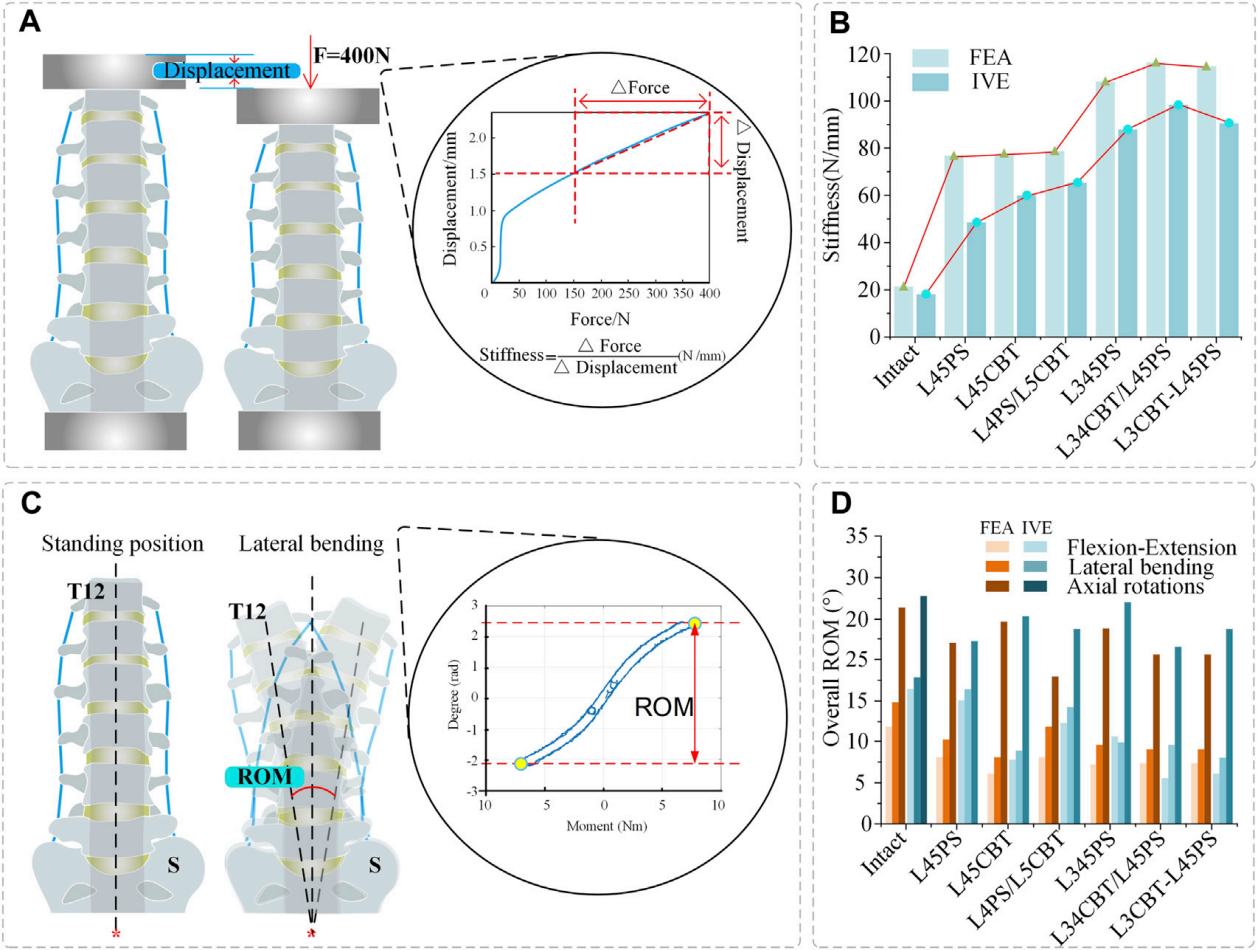
Stiffness and overall ROM. **(A)** Stiffness calculation (force–displacement curve). **(B)** Comparison of stiffness, between FEMs and IVEs under different conditions. **(C)** ROM measurement. **(D)** Comparison of overall ROM, between FEMs and IVEs under different operations.

The results of the FEM group and the IVE group showed that the stiffness of dual-segment internal fixation was stronger than that of single-segment internal fixation, and both were greater than that of the intact model, showing a specific variation pattern. The FEM group’s changing trend in single-segment internal fixation stiffness was significantly less, but the changing trend in the IVE group was clear. The results reveal that the FEM group followed the same pattern as the IVE group: L34CBT/L45PS (FEA: 115.81 N/mm, IVE: 98.12 N/mm)> L3CBT-L45PS (FEA: 114.67 N/mm, IVE: 90.44 N/mm)> L345PS (FEA: 107.77 N/mm, IVE: 87.74 N/mm)> L4CBT/L5PS (FEA: 78.27 N/mm, IVE: 65.46 N/mm)> L45CBT (FEA: 77.35 N/mm, IVE: 59.98 N/mm)> L45PS(FEA: 76.79 N/mm, IVE: 48.59 N/mm)> Intact. (Figure 5B).

### 3.2 Overall range of motion

FEM group: Compared with the intact model, the L45CBT significantly decreased ROM in flexion–extension and lateral bending conditions, but decreased ROM less in rotation. L4CBT/L5PS rotational ROM decreased the most, but ROM was decreased less in flexion and extension and lateral bending conditions. L345PS, L34CBT/L45PS, and L3CBT-L45PS displayed similar ROM performance in flexion and extension and lateral bending, and L345PS rotational ROM was less decreased. IVE group: Compared with the intact model, the overall ROM of the L45CBT was significantly reduced in flexion–extension and lateral bending conditions, but less in rotation. L45PS rotational ROM loss was significant, but ROM was decreased less in flexion and extension and lateral bending conditions. L34CBT/L45PS had a significant decrease in flexion–extension and rotational ROM. L3CBT-L45PS showed a significant decrease in ROM in lateral bending, and a slightly greater decrease in ROM in the two-segment internal fixation approach than in the single-segment internal fixation approach (Figure 5D).

### 3.3 Adjacent-segment range of motion

Following internal fixation surgery, both the FEM group and the IVE group saw varying degrees of ROM loss in adjacent-segment mobility.

**FIGURE 6:**
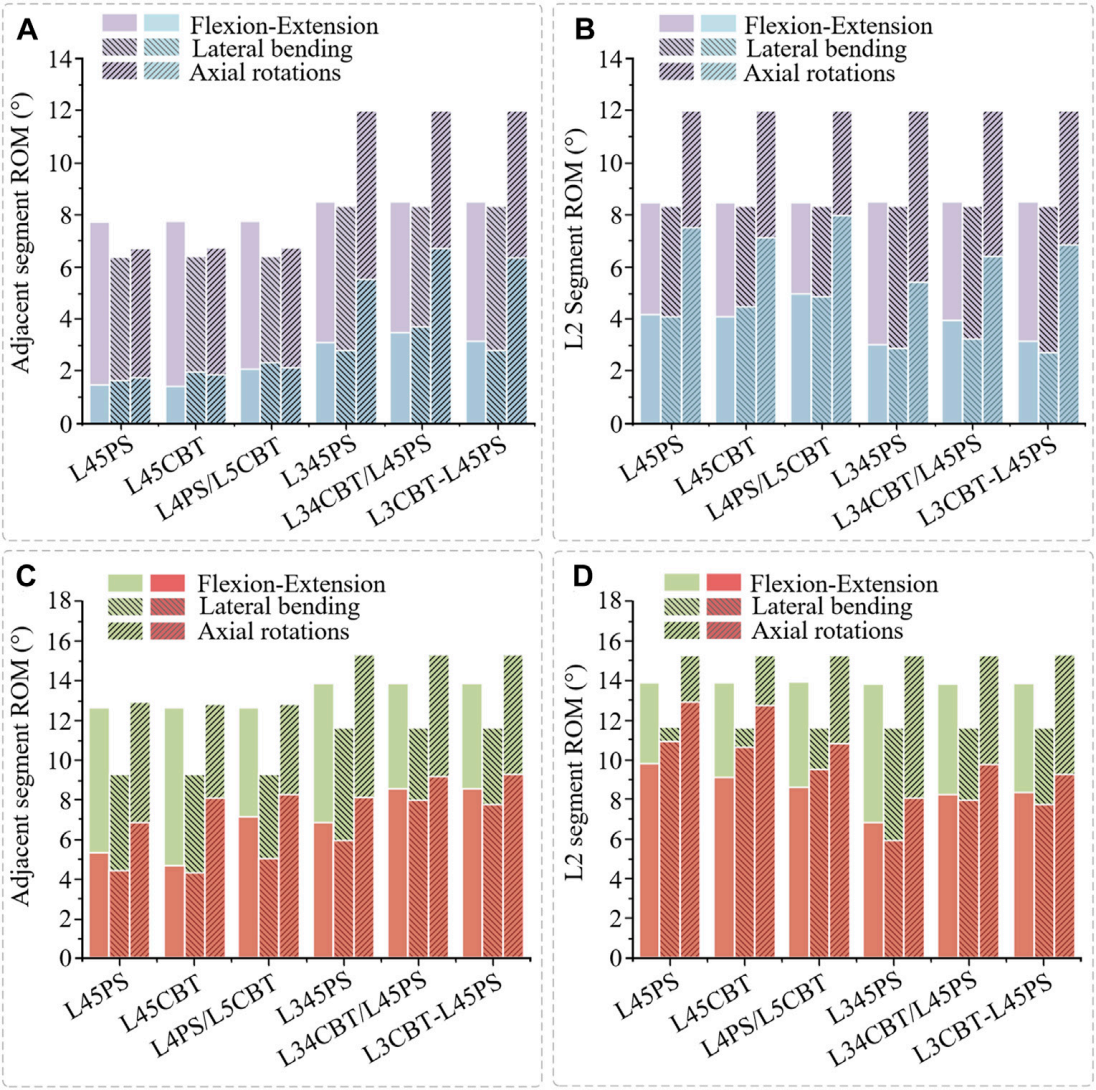
Adjacent-segment ROM and L2-segment ROM. **(A)** Comparison of adjacent-segment ROM, between postoperative FEMs (blue column) and the intact FEM (purple column). **(B)** Comparison of L2-segment ROM, between postoperative FEMs (blue column) and the intact FEM (purple column). **(C)** Comparison of adjacent-segment ROM, between postoperative IVEs (red column) and the intact IVE (green column). **(D)** Comparison of L2-segment ROM, between postoperative IVEs (red column) and the intact IVE (green column).

FEM group: Compared with the intact model, the adjacent-segment (L3) ROM of the single-segment fusion (L4-L5) was decreased. In the three conditions of flexion–extension, lateral bending, and rotational ROM, L45PS decreased by 80.1%, 73.3%, and 72.7%; L45CBT decreased by 80.9%, 70.1%, and 72.6%; and L4CBT/L5PS decreased by 72.6%, 62.5%, and 67.4%, respectively. Compared with the intact model, the adjacent-segment (L2) ROM of the dual-segment fusion (L3-L5) was decreased. L345PS decreased by 63.0%, 65.5%, and 53.7%; L3CBT-L45PS decreased by 61.6%, 65.9%, and 44.2%; and L34CBT/L45PS decreased by 54.8%, 61.8%, and 48.3% (Figure 6A).

The ROM of the adjacent segments between the single/dual-segment internal fixation is determined by the L3 segment and L2 segment, and since they are not the same segment, the single-segment internal fixation L2 segment was analyzed to facilitate a better comparison with the dual-segment internal fixation surgical approach. The outcomes demonstrated comparable changes in adjacent-segment ROM with the identical fusion segment, and the surgical technique used had no appreciable impact on adjacent-segment ROM. However, there was a difference between single-segment and dual-segment internal fixation, and dual-segment internal fixation resulted in a greater loss of adjacent-segment ROM (Figure 6B).

IVE group: Compared with the intact model, the adjacent-segment (L3) ROM of the single-segment fusion (L4-L5) was decreased. In the three conditions of flexion–extension, lateral bending, and rotation ROM, L45PS decreased by 56.0%, 52.7%, and 47.1%; L45CBT decreased by 63.8%, 55.6%, and 36.2%; and L4CBT/L5PS decreased by 44.1%, 47.5%, and 49.0%, respectively. Compared with the intact model, the adjacent-segment (L2) ROM of the dual-segment fusion (L3-L5) was decreased. L345PS decreased by 58.0%, 48.0%, and 46.8%; L3CBT-L45PS decreased by 61.1%, 61.9%, and 40.8%; and L34CBT/L45PS ROM decreased by 38.2%, 32.7%, and 37.2% (Figure 6C).

The same analysis was performed for the L2-segment ROM. L45PS decreased by 29.5%, 6.0%, and 15.4%; L45CBT decreased by 34.3%, 7.9%, and 16.7%; and L4CBT/L5PS decreased by 37.7%, 17.9%, and 33.8% (Figure 6D). With L45PS as the basis for the screw–rod lengthening procedure, L345PS, L34CBT/L45PS, and L3CBT-L45PS were decreased by 5.8%–18.7%, with a somewhat greater reduction in the ROM of IVE.

The results show that for adjacent-segment ROM, the pure CBT better preserved rotation, and the hybrid screw decreased the loss of flexion–extension and lateral bending ROM. The longer the fixed segment, the more ROM is lost.

### 3.4 Range of motion of each segment

**FIGURE 7:**
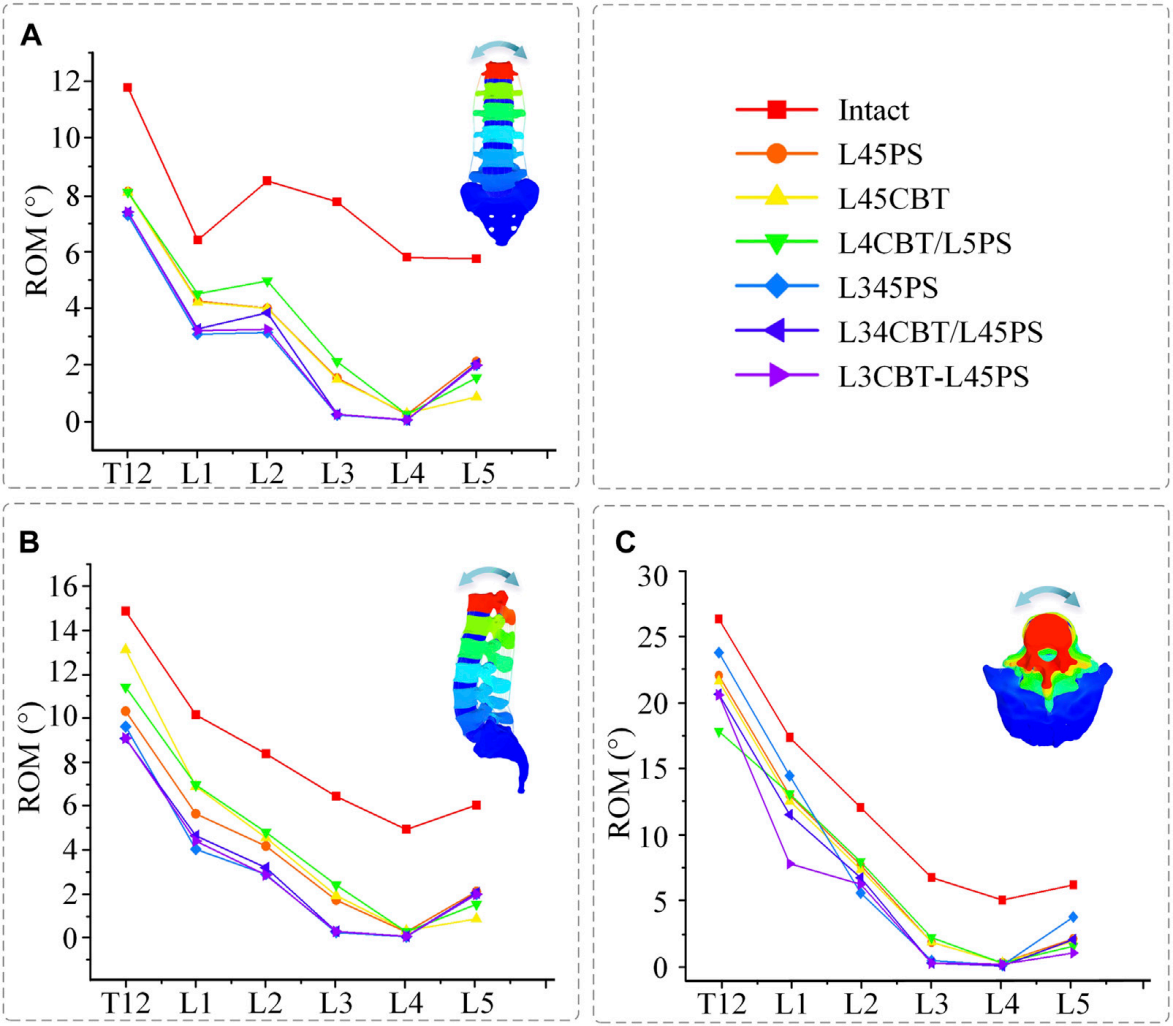
ROM for each segment between postoperative FEMs and the intact FEM. **(A)** Flexion–extension conditions. **(B)** Lateral bending conditions. **(C)** Axial rotation conditions.

For all segments of the FEM group, the ROM was read after internal fixation surgery, and as compared with the intact model, all segments’ ROM showed a decline under various conditions. For non-fixed segments, the ROM of dual-segment internal fixation is smaller than that of single-segment internal fixation under flexion–extension and lateral bending conditions. Under rotation conditions, the ROM of the model with CBT screws was found to be smaller than that of simple PS screw fixation (Figure 7).

### 3.5 Von Mises stress of the screw–rod system

**FIGURE 8:**
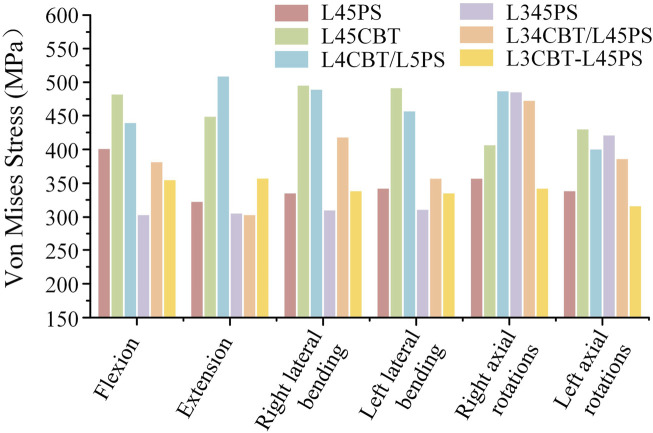
The stress value of screw–rod systems in six postoperative FEMs under different conditions.

The stress data were collected by choosing 50 points from each area of the screw–rod system stress concentration and computing the average value as the screw–rod system’s final stress value to exclude the influence of force singularities. L4CBT/L5PS and L45CBT had the highest stress values among the six conditions. L34CBT/L45PS bears more stress in rotations, L4CBT/L5PS bears more stress in extension, right lateral flexion, and left rotation, L45CBT bears more stress in flexion and left rotation, and L3CBT-L45PS bears more stress in rotation (Figure 8).

**FIGURE 9:**
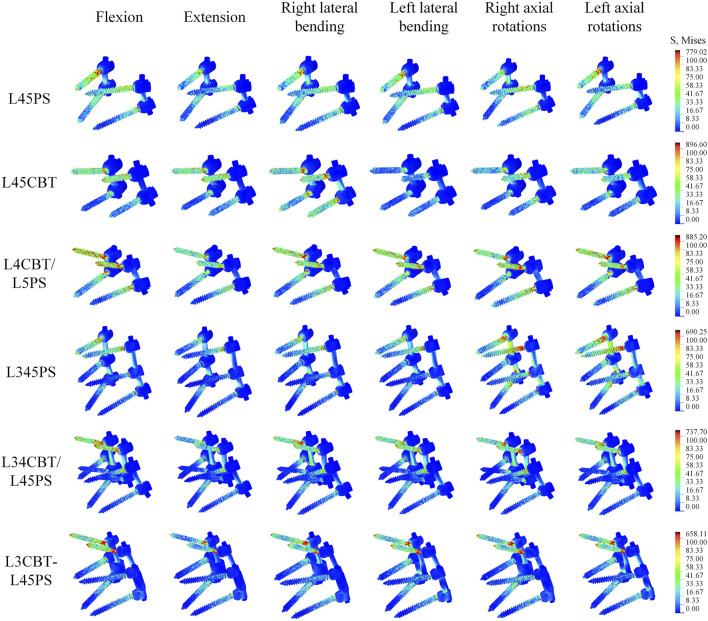
The stress distribution of screw–rod systems in six postoperative FEMs under different conditions. According to the indicator diagram, red indicates the stress concentration area, while blue shows the stress dispersion area.

The stress is mostly centered in the connecting rod’s center and the screw body’s caudal end and its precise position is where the cortical bone comes into contact with the screw, as shown by the stress cloud figure. The PS screw’s stress distribution is mostly focused in the back half of the screw body, whereas the CBT screw’s stress distribution is concentrated in the head and tail of the screw, and the entire CBT screw is under severe stress. The upper screw is significantly more stressed than the lower screw. The middle layer of three-layer screws experiences the least stress, with the majority of the stress occurring in the top and lower layer. Axial rotation considerably increases the load on the screw below. The values obtained in the studies were all well below the maximum stress values for titanium, and as a consequence, there is no risk of rupture under normal settings when analyzing the risk of fracture of the screws employed in the stabilizing system (Figure 9).

## 4 Discussion

A frequent and essential surgical approach for the treatment of spinal problems such as degenerative spine conditions is the internal fixation technique. Long and six screws both demonstrated improved lumbar spine sagittal stability. According to Spiegl et al. (2021), older individuals with mid-thoracic spinal instability treated with extended segmental stabilization had a much lower risk of subsequent vertebral fractures over time. Santoni et al. (2009) first introduced the CBT screw internal fixation approach in 2009, using screws along the caudal head sagittal and lateral paths. Later, Takata et al. (2014) presented a novel surgical technique that eliminates soft tissue stripping and shortens the incision length by fusing the upper portion with CBT screws and the lower segment with traditional pedicle screws. Numerous authors have also looked into the bone purchase of CBT screws. Using a human lumbar spine model, Santoni et al. (2009) examined the uniaxial tension of CBT screws and compared the axial pullout force of CBT screws with standard pedicle screws. Although lumbar pedicle screw fixation has the benefit of improving biomechanical stability, screw loosening and fracture can still happen as ASD progresses. The biomechanical examination of the various surgical procedures is crucial for successfully fusing the spine since it enables us to choose the best surgical strategy. There is no consensus regarding the evaluation of different spinal fusion methods leading to spinal stiffness. Additionally, little research has been conducted on the benefits and drawbacks of different fusion procedures (Park et al., 2009). In this experiment, a combination of FEA and IVE was used, and the experimental data were then cross-checked to increase their accuracy. This study’s findings provide guidance for the decision-making process in terms of the best pedicle screw therapy for ADS.

The stiffness results of the FEM group and the IVE group were similar: L34CBT/L45PS > L3CBT-L45PS > L345PS > L4CBT/L5PS > L45CBT > L45PS > Intact. This study demonstrates that hybrid screw placement and CBT may both produce greater stiffness and improved stability. The researchers also discovered that compared with short-segment fixation, long-segment fixation produced higher stiffness (Kahaer et al., 2022b). The overall stress is decreased by lengthening the internal fixation system, and several biomechanical studies have demonstrated that the use of hybrid screws enhances the biomechanical stability of the joint. The spine’s flexibility is greatly decreased by posterior fixation, and its stiffness is significantly increased. The longer the fused segment, the stiffer the spine becomes; hence, the dual-segment internal fixation strategy is stiffer than the single-segment approach. Excessive stiffness may result in excessive spinal motion and may cause ASD (Goto et al., 2003; Dai et al., 2007). At present, the term stability is misused. A stable system is one that does not undergo a large displacement under small perturbations (Liang et al., 2020). Clinically, an ROM less than 5° was considered to be a successful fusion in terms of the FDA definition (Boustani et al., 2012). Focusing only on more stiff constructions is not scientific. In order to reduce the incidence of ASD, changes in ROM must be taken into account.

In this study, the results are consistent between the FEM group and IVE group when comparing the three groups of single-segment internal fixation techniques, as determined by the total ROM loss rate. For fused segments, L45PS has high rotational resistance, whereas L45CBT has high flexion, extension, and lateral bending resistance. Comparing the three groups of two-segment internal fixation modalities, the FEM group had stronger rotational resistance with hybrid screws and similar results in the three groups for both flexion and extension and lateral bending resistance. Due to the domino connection of the screw–rod system, which leads to instability of the fused segment in the rotating state, L34CBT/L45PS and L3CBT-L45PS have greater resistance to flexion and extension as well as lateral bending in the IVE group. According to this study, L34CBT/L45PS is more effective than L3CBT-L45PS and L345PS when ASD develops as a result of single-segment fixation. Zhang et al. (2022) came to the conclusion that while the fused segment’s flexion and extension mobility were relatively low in the CBT screw group, its rotational ROM was higher than that of the PS screw, and comparable findings were found in the current investigation. Spinal flexibility is greatly decreased by posterior fixation. In a study by Elmasry et al. (2017), a posterior fixation system was discovered to have greater stiffness. According to the adjacent-segment ROM, there was no difference between the single-segment internal fixation groups and barely any difference between the dual-segment internal fixation groups. Dual-segment internal fixation had higher stability than single-segment internal fixation, but it also resulted in a substantial loss of adjacent-segment ROM. The hybrid screw design decreased the rate of ROM loss in flexion and extension and lateral bending, whereas the pure CBT treatment better maintained rotation better.

The L345PS screws were substantially more stressed than the other two groups in rotation for the dual-segment internal fixation, but the L34CBT/L45PS and L3CBT-L45PS screws were significantly more stressed than the L345PS in flexion-extension and lateral bending. For PS and CBT screws, the head and tail of the screw should be reinforced for stiffness since the stress distribution was focused in these areas where the screw made contact with the cortical bone. The upper screw stress is significantly greater than the lower screw stress, and extending the nail bar system can effectively reduce the total stress. The authors of various investigations on this subject came to the conclusion that most broken screws (78%–90%) happen in the caudal area (Chen et al., 2005; Kwon et al., 2006). Studies on the lumbar spine by Natarajan et al. revealed that the screw’s caudal area experiences the highest degree of von Mises stress, which is around 5–6 times larger than that in the screw head location and rod. (Natarajan et al., 2018). Therefore, it may be said that the rod in the same location has a higher likelihood of failing than the caudal position of the screw in posterior internal fixation.

Bone density is a risk factor for the development of postoperative ASD (Yuan et al., 2022). There is a greater surgical failure rate in patients with osteoporosis (Wu et al., 2012; Kim et al., 2015); however, osteoporosis has little effect on the ROM of the lumbar spine (Yang et al., 2009; Liu et al., 2022), so better stability should be considered when internal fixation is performed in osteoporotic patients. Our results suggest that long-segment fixation results in greater stiffness but is concomitant with a greater loss of ROM. Therefore, short-segment fixation is recommended as the first clinical option whenever possible. On this basis, CBT should be further chosen because it can provide higher stability, avoiding the occurrence of secondary operations due to the failure of screw–rod systems. Although PS is still the dominant technique for spinal internal fixation in clinical practice, CBT has a distinct advantage of reducing the incidence of ASD, thereby effectively avoiding screw–rod systems and the prolongation of surgery (Sakaura et al., 2016). Furthermore, even if a patient has developed an ASD, CBT should also be prioritized by clinicians as extended screw–rod systems, because it can provide a better retention of rotation and higher resistance to flexion, extension, and lateral bending compared with other surgical procedures. By reviewing the literature (Kahaer et al., 2022a), different scholars have conducted similar experiments with the hybrid screw and also pointed out that the hybrid screw can provide greater stability, but none of them studied the adjacent-segment ROM and did not point out the relationship with ASD development, which is also an essential highlight of this paper. The hybrid screw approach in particular has been poorly studied, and although its long-term efficacy still requires clinical validation, it is admittedly an innovation.

Both FEM and IVE methods have advantages and disadvantages. FEA has been used to analyze the biomechanical parameters within the spine and connective soft tissues that are difficult to capture by experimental techniques (Alizadeh et al., 2013; Chang et al., 2020). The use of FEA can solve some practical issues and play a significant role in clinical practice because of its relative simplicity (Wang et al., 2020b). However, due to the complexity of human structures, FEA approaches have their limitations. Different material properties and model simplifications could cause experimental results to be inaccurate. IVE can yield relatively realistic results, but its widespread use is limited by the fact that its physical specimens are scarce, expensive, and not reusable. Many scholars are currently using FEA to conduct studies, despite the inevitably great limitations of simple FEA studies; IVE is a crucial research method that will make the experimental results more accurate. Biomechanical characteristics obtained by IVE are closer to *in vitro* biomechanical characteristics (Iliescu et al., 2021). FEA and IVE are still the dominant methods for studying spinal biomechanics. In this study, FEA and specimen experiments were used to verify each other for lumbar internal fixation, and the experimental design was complete and scientific.

In this study, there are a few issues: 1) Muscles and paravertebral soft tissues were not included in the FEM and IVE, and body weight loads and muscle forces were used to calculate the loading torque. 2) The sample size should be increased to account for statistical analysis. 3) Due to the limited experimental settings, significant stress indicators were not examined in the specimen experiments. 4) The bone quality of the specimens was not taken into account for bone quality, and there were individual differences between specimens.

## 5 Conclusion

In this study, the biomechanical responses of the lumbar spine with PS and CBT fixation were investigated. Our results show that long-segment fixation produces greater stiffness than short-segment fixation, but is more likely to lead to ASD. Using a hybrid screw combination technology can considerably increase the spine’s biomechanical stability. For non-fused segments, the CBT approach provides the better retention of rotation and higher resistance to flexion, extension, and lateral bending compared with the PS technique. However, there are differences between single-segment and dual-segment internal fixation, and the ROM of adjacent segments is lost more strongly reduced by dual-segment internal fixation. Thus, the hybrid screw is an approach to consider, and is perhaps a better surgical option after clinical validation. In summary, an alternative to take into account is the hybrid screw, if internal fixation lengthening is carried out. This study can provide a better understanding of the biomechanical response to single-versus dual-segment internal fixation by different surgical procedures. A direction for future work could be to carry out statistical analysis on a larger sample of clinical data and verify the biomechanical results of this study.

## Data Availability

The raw data supporting the conclusion of this article will be made available by the authors, without undue reservation.
